# Divergent System Organ Class Safety Profiles of Isotretinoin Versus Topical Retinoids: An EudraVigilance Disproportionality Analysis

**DOI:** 10.3390/ph19010127

**Published:** 2026-01-11

**Authors:** Denisa Viola Szilagyi, Delia Mirela Tit, Ruxandra Cristina Marin, Gabriela S. Bungau, Mirela Marioara Toma, Manuela Bianca Pasca, Daniela Gitea, Laura Maria Endres

**Affiliations:** 1 Doctoral School of Biomedical Sciences, Faculty of Medicine and Pharmacy, University of Oradea, 410087 Oradea, Romania; szilagyi.denisaviola@student.uoradea.ro (D.V.S.); gbungau@uoradea.ro (G.S.B.); lendres@uoradea.ro (L.M.E.); 2 Department of Pharmacy, Faculty of Medicine and Pharmacy, University of Oradea, 410028 Oradea, Romania; toma.mirelamarioara@didactic.uoradea.ro (M.M.T.); bpasca@uoradea.ro (M.B.P.); dgitea@uoradea.ro (D.G.); 3 Department of Pharmacology, Clinical Pharmacology and Pharmacotherapy, Faculty of Medicine, “Carol Davila” University of Medicine and Pharmacy, 050474 Bucharest, Romania; 4 Department of Psycho-Neurosciences and Recovery, Faculty of Medicine and Pharmacy, University of Oradea, 410073 Oradea, Romania

**Keywords:** acne, isotretinoin, retinoids, topical retinoids, pharmacovigilance, EudraVigilance, safety, disproportionality analysis, reporting odds ratio

## Abstract

**Background/Objectives:** Isotretinoin remains an essential therapy for severe acne, yet its safety profile continues to raise concerns. This study analyzed adverse event reporting patterns for isotretinoin versus topical retinoids using EudraVigilance data. **Methods:** Aggregated ADR data for isotretinoin and four topical retinoids (tretinoin, adapalene, tazarotene, trifarotene) were retrieved from the EMA ADRreports portal (April 2025). Disproportionality was assessed using reporting odds ratios (RORs) with 95% confidence intervals at the MedDRA system organ class (SOC) level. Significant demographic differences (age and sex; both *p* < 0.001) justified stratified ROR analyses for SOCs showing positive signals. **Results:** Among 35,030 isotretinoin and 3795 topical retinoid reports, isotretinoin showed strong over-reporting in six SOCs: psychiatric disorders (ROR 11.96; 95% CI 10.11–14.14), gastrointestinal disorders (3.88; 3.50–4.31), musculoskeletal and connective tissue disorders (2.89; 2.50–3.35), surgical and medical procedures, social circumstances, and ear and labyrinth disorders. Fourteen SOCs demonstrated significant under-reporting, including neoplasms, immune system disorders, cardiac disorders, and blood/lymphatic disorders. Stratified analyses confirmed the robustness of the positive signals. Psychiatric disorders exhibited the highest disproportionality in males (22.10; 16.11–30.31) and adolescents aged 12–17 (25.85; 13.32–50.19). Gastrointestinal and musculoskeletal signals remained significant across all age and sex strata. **Conclusions:** Isotretinoin presents a distinct safety profile characterized by consistently elevated reporting of psychiatric, gastrointestinal, and musculoskeletal adverse events, independent of age and sex. These results refine the comparative safety landscape of systemic versus topical retinoids and support focused pharmacovigilance monitoring.

## 1. Introduction

A growing body of epidemiological evidence reshapes the way acne is understood in modern dermatology, not as a transient adolescent nuisance, but as a chronic inflammatory disorder with a sustained and measurable public-health impact across Europe. Recent nationwide insurance-based analyses reveal that millions of individuals seek medical care for acne each year, with German statutory health-insurance data documenting approximately 2 million affected patients in 2020. The disease peaks in late adolescence, reaching a prevalence of 13.02% at age 17, yet frequently persists into adulthood, particularly among women, where it imposes a disproportionate psychosocial burden and drives long-term healthcare utilization. Population-based data from a large Mediterranean cohort further corroborate acne persistence beyond adolescence, identifying psychosocial stressors and lifestyle factors as significant correlates of disease severity [1]. This reconceptualization of acne as a chronic, socially and clinically consequential condition has intensified scrutiny of therapeutic strategies, particularly systemic agents whose effectiveness must be balanced against safety considerations [2]. The psychosocial and quality-of-life impact of acne is substantial, including reduced self-esteem, social withdrawal, and increased risk of mood symptoms, fueling interest in early and effective treatments, especially with regard to psychiatric safety [3,4].

Current European and international treatment algorithms recommend retinoids as a cornerstone of acne management. Topical retinoids (adapalene, tretinoin, tazarotene and trifarotene) are regarded as first-line therapy for mild-to-moderate acne and as key components of maintenance regimens, either as monotherapy or in fixed combinations with benzoyl peroxide and, less frequently, topical antibiotics. Guideline-oriented reviews emphasize topical retinoids because they target follicular hyperkeratinization, normalize desquamation, and exert anti-inflammatory effects [5,6]. For more severe papulopustular or nodulocystic acne, and for moderate disease complicated by scarring or significant psychosocial distress, oral isotretinoin remains the only systemic agent recommended as monotherapy and is increasingly favored over prolonged oral antibiotics [7,8].

Recent real-world data from European health systems illustrate the scale of retinoid use. A nationwide French cohort identified over 2,020,000 dispensations of oral isotretinoin between 2019 and 2022, with 18.1% representing treatment initiations and temporary reductions during the first COVID-19 lockdown followed by recovery thereafter [9]. A complementary German claims analysis showed that topical adapalene/benzoyl peroxide accounted for approximately 86–88% of topical prescriptions, while systemic isotretinoin comprised over 80% of systemic retinoid use in both juvenile and adult acne [2]. Comparable findings outside Europe support these trends. An analysis of 8,756,594 acne-related visits in the 2018–2019 US National Ambulatory Medical Care Survey demonstrated that isotretinoin was the most frequently prescribed agent among women with acne, while topical therapies remained the mainstay of overall pharmacologic care [10].

Pharmacologically, all retinoids are vitamin A derivatives that signal through nuclear retinoic acid receptors (RAR-α, RAR-β, RAR-γ), although receptor selectivity, tissue penetration, and systemic exposure differ substantially between topical and oral formulations. Tretinoin, a first-generation retinoic acid, binds broadly to RAR isoforms and is effective for comedonal and inflammatory acne, but its irritancy and photolability have driven the development of more selective and better tolerated molecules [11].

Adapalene, a third-generation naphthoic acid derivative, was designed as a RAR-β/γ–preferring agonist and concentrates within the epidermis and dermis after topical application, with minimal systemic absorption and an excellent systemic safety profile [12,13].

Tazarotene, a synthetic prodrug converted to tazarotenic acid, also shows high affinity for RAR-β/γ. Translational and clinical data indicate that its adverse effects are predominantly local, while clinically meaningful systemic events are uncommon, consistent with very low systemic bioavailability [14].

Trifarotene, a fourth-generation topical retinoid with selective RAR-γ agonism, was developed to maximize cutaneous efficacy while minimizing systemic exposure. Pharmacokinetic and long-term safety studies demonstrate plasma concentrations in the picogram-per-milliliter range, no accumulation, and a short half-life, supporting negligible systemic absorption even under maximal application conditions [15,16,17].

In contrast, oral isotretinoin achieves sustained systemic concentrations and exerts pleiotropic effects on sebaceous gland activity, keratinocyte differentiation, and cutaneous immune signaling. These mechanisms, involving modulation of toll-like receptors, inflammatory cytokines, and sebocyte apoptosis, underlie its disease-modifying efficacy in moderate-to-severe acne [7,18].

Despite their favorable benefit–risk balance, topical retinoids are not devoid of adverse reactions. Post-2020 studies consistently report predominantly local irritant events (erythema, dryness, burning or stinging, and desquamation), while clinically meaningful systemic toxicity remains rare. Trials of fixed-combination gels containing adapalene show that most treatment-emergent events are mild, localized, and associated with low discontinuation rates and no treatment-related serious adverse events [19,20].

For tretinoin, a systematic review concluded that long-term topical use is generally safe, with adverse events dominated by mild-to-moderate erythema, peeling, burning, and dryness, and with serious or systemic reactions rarely reported [21]. A recent monograph similarly identifies photosensitivity, irritation, and xerosis as the main adverse effects in acne, with systemic toxicity largely confined to inappropriate use [22].

For tazarotene, pooled phase III data indicate that application-site pain, dryness, and exfoliation are the most common adverse events, predominantly mild and compatible with continued therapy [23]. Subsequent studies in truncal acne reported similar findings, with high patient satisfaction and no signal for clinically significant laboratory abnormalities [24].

Post-marketing analyses of trifarotene, including the PERFECT-1/2 phase III program and a 52-week safety study, document transient local irritation as the most frequent adverse effect, while treatment discontinuation is uncommon and no consistent systemic safety signal has emerged [25].

In contrast, oral isotretinoin has a broader adverse-event profile reflecting systemic exposure. Beyond mucocutaneous dryness and cheilitis, adverse reactions include transient liver enzyme elevations, hypertriglyceridemia, musculoskeletal pain, headaches, and mood symptoms. A 2024 prospective cohort study reported triglyceride or liver enzyme elevations in 35–45% of treated patients, mostly reversible with dose adjustment [8]. Neuropsychiatric effects remain under investigation, with mechanistic and clinical data suggesting potential effects on serotonergic signaling, hippocampal neurogenesis, and neuroinflammation [18]. The drug’s established teratogenic risk necessitates strict pregnancy-prevention measures worldwide [7].

Real-world evidence reinforces these safety concerns. A French nationwide cohort revealed suboptimal adherence to pregnancy-testing requirements, with only 25% of patients undergoing appropriate testing at treatment completion [26]. EudraVigilance analyses further indicate disproportionate reporting of psychiatric adverse reactions associated with isotretinoin, although causality remains debated [27].

Taken together, post-2020 clinical, mechanistic, and real-world data highlight a clear divergence between topical and oral retinoids. Topical agents are characterized by local, dose-dependent cutaneous irritation with minimal systemic risk, whereas oral isotretinoin is associated with multisystem adverse reactions, including metabolic, hepatic, musculoskeletal, neuropsychiatric, and teratogenic effects. Given their widespread use across Europe in overlapping populations, there is an urgent need to contextualize these safety signals within large, standardized pharmacovigilance datasets [2,28,29].

Given all these, there is a clear need for comparative, molecule-level pharmacovigilance data within a harmonized regulatory framework. EudraVigilance, through the EMA ADRreports interface, offers a unique opportunity to contrast the real-world safety profiles of these agents across organ systems, age groups, and sexes, using standardized MedDRA system organ classes (SOCs) as a common language. In this context, the differences in route of administration, systemic exposure, and target populations between oral isotretinoin and topical retinoids make them an informative internal comparator set to disentangle signals related to systemic pharmacology from those driven by local cutaneous toxicity and background disease.

Therefore, the present study aims to characterize and compare the SOC–level safety reporting patterns of oral isotretinoin and topical retinoids (tretinoin, adapalene, tazarotene, trifarotene) in EudraVigilance using aggregated ADR reports data and disproportionality analysis, thereby contributing to a more robust evidence base on comparative retinoid safety and offering clinically relevant information for pharmacovigilance and risk–benefit assessment.

## 2. Results

### 2.1. Descriptive Overview of Cases and Reporting Characteristics

A total of 35,030 individual case safety reports (ICSRs) were identified for isotretinoin and 3795 for topical retinoids (tretinoin, adapalene, tazarotene, trifarotene). The number of ICSRs reporting at least one adverse reaction within each MedDRA SOC was substantially higher for isotretinoin (76,045) compared with the pooled topical retinoids (8262). However, the average number of SOCs reported per ICSR was nearly identical between isotretinoin and the topical retinoid group (2.17 vs. 2.18), indicating comparable SOC-level reporting breadth across the two groups (Table 1).

Isotretinoin reports originated predominantly from adolescents and adults (12–17 years: 22.4%; 18–64 years: 49.3%), whereas topical retinoids were mostly reported in adults aged 18–64 years (57.5%) and older adults (65–85 years: 11.6%) (Table 2). A Chi-square test confirmed significant differences in age distribution between isotretinoin and topical retinoids (χ^2^(7) = 4125.24, *p* < 0.001).

Fisher’s exact tests performed for each age category showed that isotretinoin was disproportionately reported in adolescents (12–17 years), while topical retinoids were significantly over-reported in children and older adults (*p* < 0.001 for all comparisons).

Sex distribution also differed significantly (χ^2^(2) = 30.58, *p* < 0.001), with topical retinoids being more frequently reported in females and isotretinoin more common among males. Fisher’s exact tests confirmed these differences (*p* < 0.001).

Geographic origin of reports varied substantially between groups (Table 3). Isotretinoin reports originated mainly from non-EEA countries (64.0%), whereas topical retinoids showed a more balanced distribution (56.8% non-EEA).

Reporter type distribution showed the strongest divergence: topical retinoids were predominantly reported by healthcare professionals (79.6%), while isotretinoin had a much higher proportion of reports submitted by non-healthcare professionals (41.1%).

### 2.2. Seriousness Profile and System Organ Class Distribution of Reported Adverse Events

**Figure 1:**
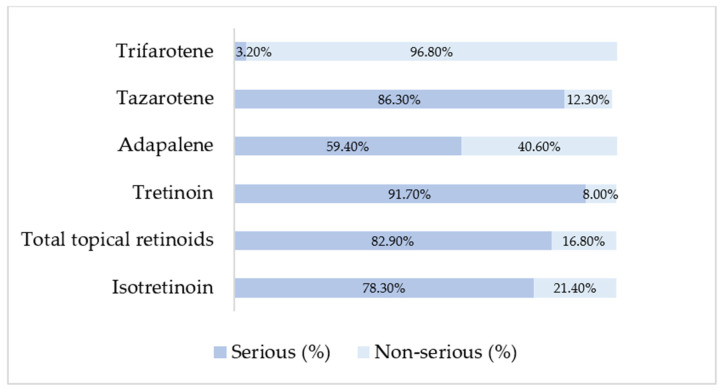
Distribution of serious and non-serious cases among retinoid products based on EMA ADRreports aggregated outputs.

The analysis of reported adverse event seriousness revealed marked differences between isotretinoin and topical retinoids (Figure 1). According to EudraVigilance reporting standards, cases were classified as serious, non-serious, or not specified. Not-specified cases represented a very small proportion for all retinoids (<0.5%), and although retained in the dataset, they are not tabulated separately due to their negligible impact on proportionality patterns. Topical retinoids overall displayed an even higher proportion of serious reports (82.9%), although substantial heterogeneity existed across individual agents (Figure 1). Tretinoin had the highest seriousness proportion (91.7% serious), consistent with its predominant reporting by healthcare professionals, while adapalene showed a more balanced profile (59.4% serious, 40.6% non-serious). Trifarotene showed the opposite pattern, with the majority of reports classified as non-serious (96.8%), reflecting its primarily localized effects and more recent post-marketing uptake.

When SOCs were ranked by descending frequency for isotretinoin, the highest number of reports were observed for gastrointestinal disorders (14.85%), closely followed by psychiatric disorders (14.76%). Skin and subcutaneous tissue disorders (8.03%), general disorders and administration site conditions (8.00%), and injury/poisoning/procedural complications (7.40%) also represented major contributors to the overall isotretinoin safety profile. These SOCs reflect the known systemic exposure associated with oral isotretinoin, including mucocutaneous toxicity, neuropsychiatric events, gastrointestinal complaints, and laboratory abnormalities (reflected under Investigations).

In contrast, topical retinoids showed SOCs distributions consistent with their localized pharmacological action. Adapalene and trifarotene were predominantly associated with skin and subcutaneous tissue disorders, whereas tretinoin exhibited a more heterogeneous pattern, with higher proportions of eye, nervous system and general disorder reports (Table 4).

Isotretinoin demonstrated a broad systemic pattern across SOCs, while topical retinoids showed narrow SOCs dominance driven by dermatologic reactions.

### 2.3. Disproportionality Analysis—Isotretinoin vs. Total Topical Retinoids

#### 2.3.1. Overall Disproportionality Analysis

Using the pooled group of all topical retinoids as comparator (TotalTRN), isotretinoin demonstrated a distinct SOC-level safety reporting profile Six SOCs showed statistically significant positive disproportionality (ROR > 1 with 95% CI fully above 1): psychiatric disorders, gastrointestinal disorders, musculoskeletal and connective tissue disorders, surgical and medical procedures, social circumstances, and ear and labyrinth disorders. Among these, psychiatric disorders showed the most pronounced signal (ROR 11.96; 95% CI 10.11–14.14), followed by gastrointestinal disorders (ROR 3.88; 95% CI 3.50–4.31).

**Figure 2:**
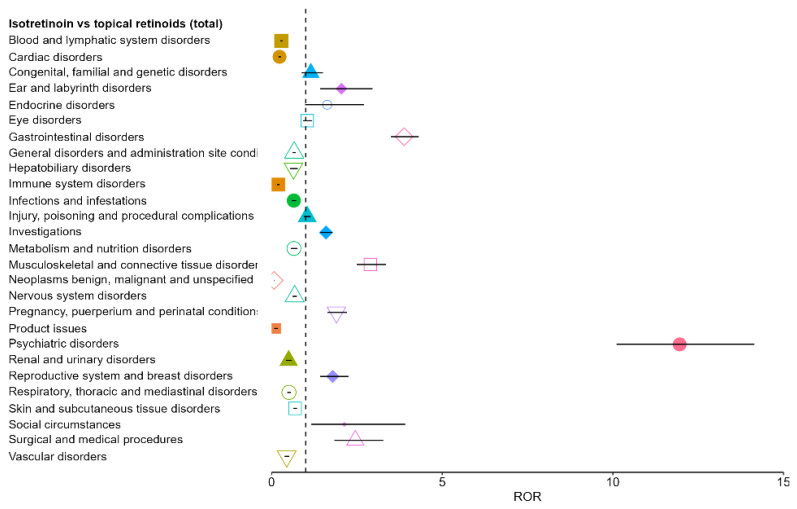
Disproportionality analysis (ROR) for isotretinoin versus all topical retinoids across SOCs. Each colored symbol (different shapes and colors) corresponds to a distinct SOC, with the marker indicating the ROR estimate and the horizontal line its 95% CI. The vertical dashed line represents the null value (ROR = 1): points to the right indicate over-reporting, while points to the left indicate under-reporting.

Conversely, isotretinoin showed significant under-reporting relative to topical retinoids across several SOCs, including neoplasms, product issues, immune system disorders, cardiac disorders, blood and lymphatic disorders, and others. Taken together, the global comparison highlights a clear over-representation of psychiatric, gastrointestinal, and musculoskeletal events for oral isotretinoin, while several systemic and topical-typical SOCs appear relatively less frequently reported. Overall, the pooled comparison indicates marked over-representation of psychiatric, gastrointestinal, and musculoskeletal events for oral isotretinoin, while multiple SOCs are relatively under-reported (Figure 2). These patterns are consistent with the expected differences between systemic and topical retinoid exposure and define the subset of SOCs carried forward to the molecule-level pairwise analyses.

#### 2.3.2. Stratified Disproportionality Analyses (Sex and Age Subgroups)

Because isotretinoin and topical retinoids showed significantly different demographic patterns in both age and sex distributions (all *p* < 0.001), the disproportionality analysis was further explored using stratified models. Stratification was limited to the three SOCs that demonstrated significant positive signals in the pooled comparison (gastrointestinal disorders, psychiatric disorders, and musculoskeletal and connective tissue disorders) and to demographic groups with adequate reporting volume (sex: male, female; age: 12–17 years, 18–64 years). No reliable stratification could be performed for younger children (<12 years) or older adults (≥65 years) because of sparse isotretinoin reporting, and negative disproportionality signals were not stratified, in line with EMA recommendations to avoid unstable estimates.

Isotretinoin showed stronger disproportionality signals in males across all three SOCs. For psychiatric disorders, the ROR reached 22.10 (95% CI 16.11–30.31) in males compared with 8.63 (95% CI 7.01–10.63) in females. For gastrointestinal disorders, males showed an ROR of 5.59 (95% CI 4.71–6.64) versus 2.89 (95% CI 2.52–3.31) in females. For musculoskeletal disorders, males exhibited an ROR of 4.11 (95% CI 3.19–5.31), whereas females had an ROR of 2.37 (95% CI 1.96–2.86). These findings suggest that sex acts as an effect modifier, with consistently larger disproportionality estimates in males.

Stratification by age also confirmed the robustness of the signals. For psychiatric disorders, the disproportionality was particularly strong among adolescents (12–17 years: ROR 25.85, 95% CI 13.32–50.19), compared with adults (18–64 years: ROR 11.70, 95% CI 9.55–14.33). In contrast, gastrointestinal disorders demonstrated similar disproportionality in both age strata (12–17 years: 4.36; 18–64 years: 4.24). For musculoskeletal disorders, adolescents exhibited a notably stronger signal (ROR 7.38, 95% CI 3.92–13.87) than adults (ROR 2.57, 95% CI 2.16–3.05).

**Figure 3:**
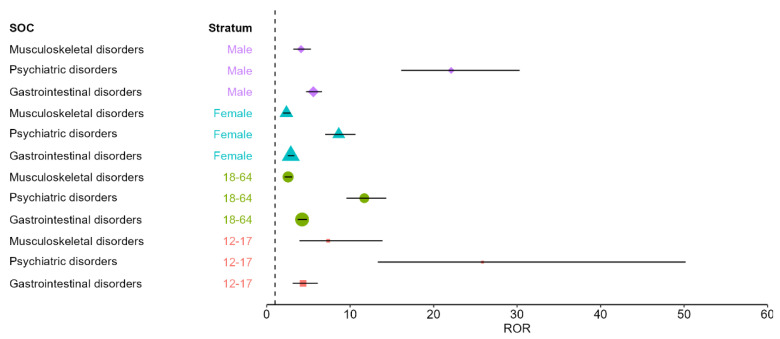
Stratified disproportionality (ROR) for isotretinoin versus pooled topical retinoids across SOCs with positive signals, by age group and sex. Symbols indicate stratum-specific ROR estimates and horizontal bars the 95% CIs. The vertical dashed line represents the null value (ROR = 1); values > 1 indicate over-reporting for isotretinoin relative to topical retinoids.

Overall, the stratified analyses confirm that isotretinoin’s core disproportionality profile is robust across the main demographic strata, with psychiatric disorders showing the highest signal and with consistently larger estimates in males and adolescents (Figure 3).

### 2.4. Pairwise Comparisons Between Isotretinoin and Individual Topical Retinoids

**Figure 4:**
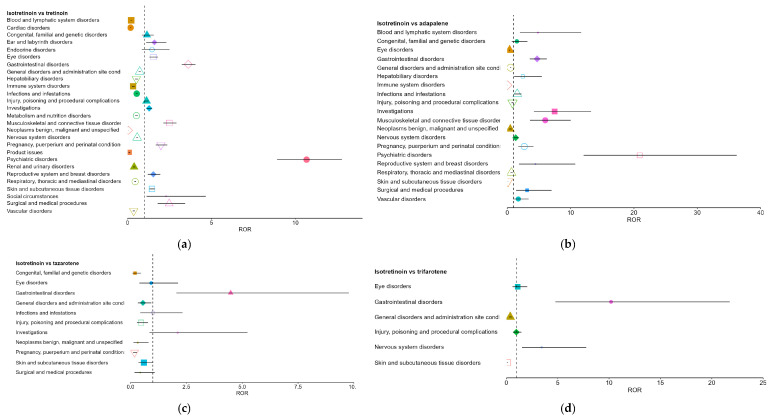
Pairwise comparisons between isotretinoin and individual topical retinoids. (**a**). Isotretinoin vs. tretinoin; (**b**). Isotretinoin vs. adapalene; (**c**). Isotretinoin vs. tazarotene; (**d**). Isotretinoin vs. trifarotene. Each colored symbol (different shapes and colors) corresponds to a distinct stratum, with the marker indicating the ROR estimate and the horizontal line its 95% CI. The vertical dashed line represents the null value (ROR = 1): points to the right indicate over-reporting, while points to the left indicate under-reporting. Estimates for tazarotene and trifarotene are based on fewer reports and therefore have wider confidence intervals.

Following the pooled comparison, pairwise analyses were conducted to explore molecule-specific differences between isotretinoin and each topical retinoid (tretinoin, adapalene, tazarotene, and trifarotene). Pairwise results are presented as supportive to the pooled analysis, and interpretation focuses on the SOCs driving the main pooled signals. The full set of pairwise RORs is shown in Figure 4a–d. Overall, the pairwise comparisons were directionally consistent with the class-level results. Across comparators, isotretinoin showed prominent over-reporting in psychiatric disorders, gastrointestinal disorders, and musculoskeletal and connective tissue disorders, although effect sizes varied.

Against tretinoin, isotretinoin demonstrated broad over-reporting signals, with RORs of 10.67 for psychiatric disorders, 3.61 for gastrointestinal disorders, and 2.49 for musculoskeletal disorders. Several SOCs also showed significant under-reporting, notably cardiac and hematologic disorders (Figure 4a).

In comparison with adapalene, isotretinoin exhibited even larger effect sizes, particularly for psychiatric (ROR 20.90) and musculoskeletal disorders (ROR 6.00). Conversely, SOCs typical of topical exposure, such as skin and administration-site disorders, showed marked under-reporting for isotretinoin (RORs < 1) (Figure 4b).

For tazarotene and trifarotene (Figure 4c,d), analyses were limited by small sample sizes, yet the same directional trends persisted: isotretinoin showed over-reporting for gastrointestinal disorders and under-reporting for general and skin-related SOCs. Although less stable, these estimates supported the general pattern observed in larger comparator groups.

### 2.5. Cross-Retinoid Comparison of Disproportionality Signals

Across pairwise disproportionality analyses, psychiatric, gastrointestinal, and musculoskeletal and connective tissue disorders emerged as the SOCs most consistently and strongly over-reported for isotretinoin relative to all topical retinoids. These associations frequently showed large effect sizes, often exceeding one order of magnitude for psychiatric disorders. In contrast, immune system, cardiac, blood and lymphatic, and general/administration site disorders were repeatedly under-reported for isotretinoin, a pattern coherent with the systemic versus topical pharmacological profiles of the compared agents.

The pooled isotretinoin-versus-topical-retinoids analysis amplified these trends, with psychiatric disorders representing the most prominent signal (ROR = 11.96), followed by gastrointestinal and musculoskeletal SOCs. Pairwise comparisons with individual topical retinoids reproduced these patterns with varying magnitudes, confirming the stability of the isotretinoin signal across comparators.

A complete overview of all statistically significant SOC-level ROR estimates (including 95% confidence intervals) is presented in Table 5, summarizing both positive and negative disproportionality signals across all comparator retinoids.

## 3. Discussion

By integrating global, stratified, and pairwise disproportionality analyses, this study aimed to provide a nuanced, clinically interpretable map of psychiatric and systemic safety signals for isotretinoin relative to topical retinoids in European pharmacovigilance data. This disproportionality analysis revealed distinct pharmacovigilance signal profiles for oral isotretinoin compared with topical retinoids, reflecting both expected pharmacological differences and additional reporting patterns related to demographics and reporter types, underscoring the value of spontaneous reporting data in complementing pre-authorization trials [30]. By selecting topical retinoids, agents with similar dermatologic indications but minimal systemic exposure, as an active comparator, the analysis sought to disentangle drug-specific systemic risks from the background morbidity and psychosocial burden of acne [31,32]. The use of spontaneous reports also enabled exploration of age, sex, geographic origin, and reporter-type patterns not captured in randomized trials but increasingly recognized as modifiers of isotretinoin’s benefit–risk balance [29,33].

From a descriptive standpoint, isotretinoin accounted for a far greater number of ICSRs and SOC-linked case reports compared to all topical retinoids combined. This aligns with its role as a widely used systemic treatment for moderate-to-severe acne [34]. However, the mean number of SOCs reported per ICSR was almost identical between isotretinoin and topical retinoids (2.17 vs. 2.18), suggesting that the breadth of organ system involvement per case was comparable across products. These results are consistent with existing pharmacovigilance literature showing that the completeness of reports primarily depends on the quality and depth of clinical information provided per case [35].

Clear demographic differences emerged. Isotretinoin reports were concentrated among adolescents and young adults, whereas topical retinoid reports more often involved older patients, mirroring real-world prescribing patterns [31,34]. Guidelines emphasize isotretinoin for severe adolescent acne and topical retinoids for milder disease and maintenance across age groups [29].

Accordingly, age distribution differences were highly significant (*p* < 0.001), with isotretinoin over-represented in adolescents and topical retinoids more frequent in children and older adults, potentially reflecting off-label or non-acne use of topical agents. Sex distribution also differed significantly (*p* < 0.001). Isotretinoin reports showed slight male predominance, while topical retinoid reports were more often female, consistent with prescribing trends and teratogenic risk considerations. A large cohort study demonstrated that male sex more than doubled the odds of isotretinoin use [34], whereas females more frequently use topical retinoids for acne and cosmetic indications [17,31].

Geographic patterns diverged as well. Most isotretinoin ICSRs originated from outside the EEA, whereas topical retinoid reports were more evenly distributed, consistent with previous analyses of isotretinoin psychiatric adverse events [27]. These differences may reflect variability in access, regulatory frameworks, cultural attitudes, and reporting awareness [36]. In contrast, the balanced distribution for topical retinoids aligns with their widespread first-line use and favorable systemic safety profile [31].

Reporter type further distinguished the two groups. Isotretinoin reports included a substantial proportion of non-healthcare professionals, whereas topical retinoid reports were predominantly submitted by healthcare professionals. This likely reflects isotretinoin’s public visibility and concern regarding systemic and psychiatric effects. Consumer reports now account for roughly one-third of European ADRs submissions and can meaningfully contribute to signal detection, with comparable completeness to professional reports [33,35].

Thus, the high fraction of patient-reported isotretinoin cases in our data is not only indicative of greater patient engagement (and concern) with this drug but also suggests that the signals gleaned from such reports are credible. In contrast, the adverse effects of topical retinoids (like skin irritation) are often mild and might be addressed in routine office visits, which could explain why healthcare professionals dominate the reporting for those drugs, typical minor retinoid reactions might not prompt patients to file reports themselves.

Regarding seriousness, most isotretinoin reports were classified as serious (78.3%), consistent with its known systemic adverse-effect spectrum [18,37]. Although topical retinoids overall showed a high proportion of serious cases, this was driven largely by tretinoin, whereas adapalene and trifarotene displayed more balanced or predominantly non-serious profiles. These findings suggest that mild topical reactions are less likely to be reported, while isotretinoin reports capture both routine and severe outcomes. Similar real-world analyses have highlighted isotretinoin’s association with severe outcomes such as hepatotoxicity, inflammatory bowel disease flares, and mood disturbances [30].

The benign profile observed for trifarotene aligns with clinical trial data showing mainly mild local reactions and virtually no serious treatment-related events [38,39]. Likewise, adapalene’s safety profile corresponds with trial evidence of predominantly local irritation and very low systemic risk [19,40].

Consistent with these differences, the distribution of adverse reactions across SOCs was much broader for isotretinoin. The most frequent SOCs were gastrointestinal and psychiatric disorders, followed by skin, general disorders, and injury-related categories, reflecting isotretinoin’s multi-system effects [30]. Psychiatric disproportionality signals, including depressive and mood disorders, have been consistently reported, particularly among pediatric and adolescent patients [36,41]. Although population-based studies do not demonstrate an increased long-term risk of diagnosed psychiatric disorders or suicide, pharmacovigilance data emphasize that clinically significant psychiatric reactions occur in susceptible individuals [42,43].

Musculoskeletal and investigation-related SOCs were also prominent for isotretinoin, in line with known effects on lipids, liver enzymes, and musculoskeletal symptoms [18,44,45]. In contrast, SOC patterns for topical retinoids largely reflected local dermatologic reactions with minimal systemic involvement [38,46]. Studies of fixed-dose topical combinations further support the confinement of topical retinoid risk to skin-related SOCs [40].

Although tretinoin showed relatively higher proportions of nervous system and eye disorders, its overall systemic safety profile remains favorable [47]. No topical retinoid demonstrated the multi-organ involvement characteristic of isotretinoin.

To formally quantify these differences, we performed disproportionality analysis using RORs comparing isotretinoin to topical retinoids. In the overall analysis (pooling all topical retinoids as the comparator), isotretinoin showed statistically significant over-reporting in several SOCs, most strikingly in psychiatric, gastrointestinal, and musculoskeletal events. The ROR for psychiatric disorders was approximately 12, indicating that reports including at least one psychiatric ADR were about twelve times more likely for isotretinoin than for topical retinoids, consistent with disproportionate reporting of depression, anxiety, mood changes, and suicidal ideation in pharmacovigilance databases [27,48].

For gastrointestinal disorders, the overall ROR was around 3.9, meaning isotretinoin had nearly four times the reporting odds of gastrointestinal events compared to topicals, in line with signal-mining studies linking isotretinoin to gastrointestinal problems including inflammatory bowel disease flares and other gastrointestinal symptoms, although causality remains debated [30,49].

Musculoskeletal and connective tissue disorders were also significantly over-reported (pooled ROR 2.9), echoing reports of musculoskeletal pain and rarer effects such as premature epiphyseal closure or hyperostosis [44].

Beyond these top three signals, a few other SOCs had smaller but significant ROR > 1 for isotretinoin, including surgical and medical procedures and social circumstances, likely reflecting downstream consequences reported in association with severe ADRs and impacts on functioning [50]. A modest positive disproportionality was also observed for ear and labyrinth disorders (overall ROR 2.1), corresponding to infrequently reported effects such as tinnitus or hearing loss [51,52].

Conversely, isotretinoin was significantly under-reported (ROR < 1) relative to topical retinoids in SOCs including neoplasms, cardiac, blood and lymphatic, and immune system disorders. These findings align with clinical evidence not supporting a substantial increase in overall cancer or major cardiovascular event rates attributable to isotretinoin in acne populations [53,54]. Similarly, although isotretinoin can induce dyslipidemia and transient laboratory abnormalities, recent prospective studies indicate these changes are usually mild and reversible and have not translated into an excess of clinically manifest thromboembolic or hematologic events at the population level, consistent with RORs below unity in blood and lymphatic SOCs compared with topical retinoids [55].

Taken together, our SOC-level disproportionality results support a characteristic isotretinoin safety signal architecture dominated by psychiatric, gastrointestinal, and musculoskeletal events, which is not shared by topical retinoids and aligns with mechanistic considerations and real-world safety data.

Because isotretinoin and topical retinoid populations differed in age and sex, we explored key disproportionality signals within more homogeneous subgroups. Stratified analyses confirmed persistence of psychiatric, gastrointestinal, and musculoskeletal over-reporting across sexes and across adolescents and adults, with differences in magnitude. In sex-stratified models, RORs for all three major SOC signals were higher in males than females, with a particularly elevated psychiatric ROR in males (point estimate over 20 vs. about 8.6 in females). This indicates disproportionately higher reporting of psychiatric ADRs in male isotretinoin reports relative to topical retinoids and may reflect differences in symptom recognition or reporting behavior, as well as residual confounding [36].

Retrospective studies have also noted that some severe isotretinoin adverse effects, including musculoskeletal conditions such as sacroiliitis or inflammatory arthritis, appear more frequent in male patients [56], which could partly explain the higher musculoskeletal ROR observed in males.

When stratifying by age, the adolescent subgroup (12–17 years old) exhibited the strongest disproportionality signals. The psychiatric disorder ROR in adolescents was on the order of 25 or higher, exceeding the elevated ROR of 11 among adults 18–64, consistent with prior pharmacovigilance observations that serious psychiatric ADR reports are frequently concentrated in teenagers and young adults despite uncertain causality at the population level [42,57]. Similarly, the musculoskeletal signal was stronger in adolescents (ROR 7.4) than adults (2.6), potentially reflecting greater susceptibility of growing patients, consistent with evidence linking isotretinoin to premature epiphyseal closure and exacerbation of back pain or inflammatory spinal pain in adolescents [56,58]. In contrast, gastrointestinal disproportionality was similar in adolescents and adults (both around ROR 4), suggesting that factors such as cumulative dose or predisposition may be more relevant than age [49].

Overall, these analyses indicate that isotretinoin’s core safety signals are robust across demographics, with amplification in groups heavily exposed to isotretinoin or potentially more biologically vulnerable. Clinically, this supports careful monitoring of mood symptoms and musculoskeletal complaints in teenagers and attention to sex-specific factors during isotretinoin therapy [36].

In addition to the pooled analysis, we conducted head-to-head disproportionality comparisons between isotretinoin and each topical retinoid. These comparisons echoed the class-level findings while showing molecule-specific differences. Against tretinoin, isotretinoin showed over-reporting in psychiatric (ROR 10.7) and gastrointestinal SOCs (ROR 3.6), and higher musculoskeletal reporting (ROR 2.5). Versus adapalene, effect sizes were larger, with psychiatric ROR 20.9 and musculoskeletal ROR 6.0, consistent with adapalene’s excellent systemic safety record [13,38,59].

Isotretinoin was also under-reported for local irritant effects in these comparisons; for example, the ROR for skin and subcutaneous tissue disorders was 0.11 versus adapalene, reflecting common topical irritation with adapalene and the absence of application-site reactions with oral isotretinoin. Similarly, in the isotretinoin versus trifarotene comparison, isotretinoin showed higher odds of gastrointestinal events but under-reporting for skin irritation (ROR for skin disorders < 0.1 vs. trifarotene). Comparisons with tazarotene were limited by small case numbers but followed the same direction, with more systemic signals for isotretinoin and fewer local reactions.

These pairwise results support attribution of the observed differences to isotretinoin’s systemic pharmacology rather than database idiosyncrasies. Notably, topical retinoids did not show the psychiatric signal prominent for isotretinoin, consistent with regulatory assessments indicating no association between topical retinoids and depression or suicide risk [60].

The persistence of isotretinoin signals across comparators, including older (tretinoin) and newer (trifarotene) agents, supports a class-level contrast between systemic and primarily local-acting retinoids.

This study has several methodological limitations that warrant consideration when interpreting the findings. It relies on spontaneous reports from the EudraVigilance database, as accessed through the EMA ADRreports portal, which is subject to well-known constraints such as underreporting, differential reporting by country and reporter type, and stimulated reporting following media attention or regulatory action. The use of aggregated data precluded validation of individual case narratives and limited our ability to examine dose, treatment duration, indication details, comorbidities, and concomitant medications, all of which may confound the observed associations. Furthermore, the absence of reliable exposure denominators means that we could assess reporting disproportionality rather than absolute incidence or risk, and causal inference cannot be established. Differences in market duration, utilization patterns, and age/sex distributions between isotretinoin and individual topical retinoids may also contribute to residual confounding despite the use of stratified analyses.

A particularly important limitation concerns MedDRA granularity. Disproportionality was conducted at the SOC level, which is broad and clinically heterogeneous. SOC-level signals do not identify which preferred terms (PT) drive the observed disproportionality; therefore, results should be interpreted as a screening map of organ-system domains rather than as evidence for specific adverse reactions. Some topical comparators, particularly newer agents such as trifarotene, had small numbers of reports, yielding wider confidence intervals and less precise estimates. The exploratory design and lack of formal adjustment for multiple testing also increase the possibility of chance findings, especially for marginal signals. PT-level analyses and standardized MedDRA query-based assessments using case-level data are warranted to clarify which specific reactions underlie the strongest SOC signals and to reduce within-SOC heterogeneity.

Despite these limitations, the study has several notable strengths that enhance the robustness and clinical relevance of its conclusions. By selecting topical retinoids as an active comparator group sharing similar dermatologic indications but minimal systemic exposure, the analysis more effectively isolates safety signals that are likely to be specific to systemic isotretinoin rather than to acne itself or its psychosocial burden. The large number of ICSRs, the comprehensive inclusion of all available reports without temporal or geographic restriction, and the combination of pooled, stratified, and pairwise disproportionality analyses provide a coherent and internally consistent picture of isotretinoin’s safety signal architecture. The convergence of findings across different comparators and demographic strata, particularly for psychiatric, gastrointestinal, and musculoskeletal SOCs, supports the robustness of the signals and their plausibility in light of isotretinoin’s pharmacology. Moreover, leveraging EudraVigilance allowed exploration of age, sex, geographic origin, and reporter-type patterns that are typically underrepresented or absent in randomized trials and cohort studies, thereby complementing existing evidence. Taken together, these strengths suggest that, even within the constraints of spontaneous reporting data, the present analysis offers a methodologically transparent and clinically informative contribution to understanding the differential safety profiles of systemic versus topical retinoid therapies.

## 4. Materials and Methods

### 4.1. Data Source

We utilized publicly available data from the European Medicines Agency’s ADRreports portal, which provides aggregated EudraVigilance reports [61]. EudraVigilance is the EU pharmacovigilance database that collects spontaneous adverse event reports from member countries and beyond. The ADRreports web interface (accessed on 20 April 2025) allows queries by drug/substance name, returning summary counts of ICSRs stratified by various categories (age group, sex, reporter type, geographic origin), including a breakdown by reported SOCs of reactions. Each ICSR corresponds to a unique case report of one or more adverse reactions linked to a suspect drug. For this study, we extracted data on isotretinoin and on four topical retinoid active substances: tretinoin, adapalene, tazarotene, and trifarotene. No filters were applied; all ICSRs in the database for each substance were included.

### 4.2. Study Design

The study compared the spectrum of reported adverse events between oral isotretinoin and topical retinoids at the SOC level. This level was selected because the ADRreports portal provides publicly accessible aggregated EudraVigilance outputs that are primarily structured by SOC, enabling stable and reproducible cross-product comparisons within a high-level screening framework. The unit of analysis was the ICSR. Within each SOC, reports were counted once per ICSR based on the presence or absence of any reaction mapped to that SOC. As a result, a single ICSR may contribute to multiple SOCs, reflecting multi-system reporting, while duplicate counting within the same SOC is avoided.

A pooled comparison (isotretinoin vs. all topical retinoids combined) was first conducted to obtain an overall class-level safety signal evaluation. Subsequently, pairwise comparisons of isotretinoin with each topical retinoid (i.e., isotretinoin vs. tretinoin, isotretinoin vs. adapalene, etc.) were performed to identify molecule-specific signal variations. These pairwise analyses were restricted to SOCs that showed a significant signal in the pooled isotretinoin-vs-topicals comparison.

Because isotretinoin and topical retinoids showed significant differences in age and sex reporting distributions (all *p* < 0.001), additional stratified disproportionality analyses were performed to assess the robustness of the main positive signals. Stratified RORs were computed only for SOCs with significant positive disproportionality in the pooled analysis (psychiatric, gastrointestinal, and musculoskeletal disorders), and only within strata with sufficient reporting volume to ensure stable estimates. Stratification was conducted by sex (male, female) and by age (12–17 years and 18–64 years), while younger children and older adults were not stratified due to sparse isotretinoin reports. Negative disproportionality signals (ROR < 1) were not stratified, as these do not represent safety signals and subgroup analyses may yield unstable estimates in spontaneous-reporting data.

### 4.3. Disproportionality Method

To identify potential safety signals, we applied a standard disproportionality approach using the reporting odds ratio (ROR). The ROR estimates the relative likelihood of a given SOC being reported for isotretinoin compared to a reference drug or group. For each comparison, the 2 × 2 contingency table [62] was constructed from the number of reports with and without the SOC for both isotretinoin and the comparator.

Confidence intervals (95%) were calculated using the log-transformed standard error of the ROR. Only comparisons where all four cells of the contingency table contained at least five reports were considered valid and included in the final analysis. A ROR > 1 with a 95% CI entirely above 1.0 was interpreted as a positive signal, while a ROR < 1 with a CI entirely below 1.0 was interpreted as a negative signal, possibly indicating reduced reporting [63].

### 4.4. Statistical Considerations

The analysis was descriptive and exploratory in nature. No corrections for multiple testing were applied. All calculations were performed in Microsoft Excel and JASP (v0.19.3). MedDRA SOCs were used without modification, and no attempt was made to reclassify or group terms beyond those presented in the ADRreports portal. PT-level analyses were not undertaken in this study to preserve a consistent SOC-level screening framework based on aggregated outputs; therefore, SOC signals are interpreted as broad organ-system reporting patterns rather than specific adverse reactions.

## 5. Conclusions

This comparative pharmacovigilance analysis showed that isotretinoin was consistently associated with disproportionate reporting of psychiatric, gastrointestinal, and musculoskeletal adverse events, whereas topical retinoids were largely confined to dermatologic and local intolerance reactions. The persistence of key signals across demographic strata suggests that population characteristics may influence the expression or reporting of adverse events. Conversely, the lack of positive disproportionality in several systemic SOCs aligns with current clinical evidence and provides reassurance regarding those outcomes when isotretinoin is prescribed appropriately.

Clinically, these results underscore the need for focused surveillance of mood changes, gastrointestinal symptoms, and musculoskeletal complaints in patients receiving systemic retinoids, while reaffirming the largely local and benign safety profile of topical retinoids. Further research incorporating exposure-adjusted data and detailed clinical characterization will be important for strengthening patient-level risk stratification for systemic retinoid therapy.

## Figures and Tables

**Table 1:** Reporting characteristics for isotretinoin and topical retinoids in EudraVigilance.

Retinoid	SOC-Linked ICSRs	ICSR	SOCs per ICSR
Isotretinoin	76,045	35,030	2.17
Total topical retinoids	8262	3795	2.18
Tretinoin	6814	2976	2.29
Adapalene	1200	589	2.04
Tazarotene	154	73	2.11
Trifarotene	230	157	1.47

SOC, system organ class; ICSR, individual case safety report. SOC-linked ICSRs represent the number of ICSRs reporting at least one adverse reaction coded under a given SOC. Each ICSR is counted once per SOC, regardless of the number of reactions recorded within that SOC. SOCs per ICSR refers to the mean number of SOCs reported per case. Despite the higher overall reporting volume for isotretinoin, the mean SOCs per ICSR was similar for isotretinoin and pooled topical retinoids.

**Table 2:** Distribution of ICSRs by age group and sex for all retinoids analyzed (including total topical retinoids).

Category	Isotretinoin	Tretinoin	Adapalene	Tazarotene	Trifarotene	Total Topical Retinoids
Age group, n (%)						
Not specified	9342 (26.7)	374 (12.6)	139 (23.6)	33 (45.2)	61 (38.9)	607 (16.0)
0–1 month	171 (0.5)	14 (0.5)	4 (0.7)	1 (1.4)	0 (0.0)	19 (0.50)
2 months–2 years	75 (0.2)	36 (1.2)	0 (0.0)	1 (1.4)	0 (0.0)	37 (0.97)
3–11 years	178 (0.5)	136 (4.6)	4 (0.7)	1 (1.4)	0 (0.0)	141 (3.7)
12–17 years	7860 (22.4)	181 (6.1)	138 (23.4)	3 (4.1)	26 (16.6)	348 (9.2)
18–64 years	17,276 (49.3)	1792 (60.2)	289 (49.1)	30 (41.1)	70 (44.6)	2181 (57.5)
65–85 years	125 (0.4)	423 (14.2)	13 (2.2)	3 (4.1)	0 (0.0)	439 (11.6)
>85 years	3 (0.0)	20 (0.7)	2 (0.3)	1 (1.4)	0 (0.0)	23 (0.6)
Total	35,030	2976	589	73	157	3795
Sex, n (%)						
Female	17,852 (51.0)	1513 (50.8)	435 (73.9)	52 (71.2)	106 (67.5)	2106 (55.5)
Male	15,131 (43.2)	1263 (42.4)	134 (22.8)	18 (24.7)	50 (31.8)	1465 (38.6)
Not specified	2047 (5.8)	200 (6.7)	20 (3.4)	3 (4.1)	1 (0.6)	224 (5.9)
Total	35,030	2976	589	73	157	3795

Percentages are column-wise. Distributions indicate younger age concentration for isotretinoin (12–17; 18–64), a higher older-adult share for tretinoin (65–85), and a stronger female predominance for topical retinoids (especially adapalene). Missing age proportions varied by product.

**Table 3:** Geographic and reporter-type characteristics.

Category	Isotretinoin	Tretinoin	Adapalene	Tazarotene	Trifarotene
	n (%)				
Geographic region					
European Economic Area	12,615 (36.0)	1173 (39.4)	301 (51.1)	12 (16.4)	154 (98.1)
Non-European Economic Area	22,414 (64.0)	1803 (60.6)	288 (48.9)	61 (83.6)	3 (1.9)
Not specified	1 (0.0)	0 (0.0)	0 (0.0)	0 (0.0)	0 (0.0)
Reporter type					
Healthcare professional	20,544 (58.6)	2666 (89.6)	202 (34.3)	49 (67.1)	97 (61.8)
Non-healthcare professional	14,391 (41.1)	304 (10.2)	387 (65.7)	23 (31.5)	60 (38.2)
Not specified	95 (0.3)	6 (0.2)	0 (0.0)	1 (1.4)	0 (0.0)

**Table 4:** Distribution of ICSRs across SOCs for isotretinoin and topical retinoids, ordered by decreasing isotretinoin frequency.

SOC	Isotretinoin	Tretinoin	Adapalene	Tazarotene	Trifarotene
	n (%)				
Gastrointestinal disorders	11290 (32.23)	346 (11.63)	54 (9.17)	7 (9.59)	7 (4.46)
Psychiatric disorders	11226 (32.05)	126 (4.23)	13 (2.21)	4 (5.48)	1 (0.64)
Skin and subcutaneous disorders	6107 (17.43)	377 (12.67)	385 (65.38)	19 (26.03)	110 (70.06)
General disorders and administration site	6082 (17.36)	671 (22.55)	169 (28.69)	20 (27.40)	57 (36.31)
Injury, poisoning and procedural complications	5626 (16.06)	430 (14.45)	111 (18.85)	21 (28.77)	26 (16.56)
Musculoskeletal and connective tissue disorders	4745 (13.55)	176 (5.91)	15 (2.54)	4 (5.48)	0 (0.00)
Investigations	4709 (13.44)	320 (10.75)	12 (2.04)	5 (6.85)	0 (0.00)
Nervous system disorders	4206 (12.01)	573 (19.25)	55 (9.34)	4 (5.48)	6 (3.82)
Pregnancy, puerperium and perinatal	3157 (9.01)	141 (4.74)	21 (3.56)	26 (35.62)	0 (0.00)
Infections and infestations	2902 (8.28)	423 (14.21)	32 (5.43)	6 (8.22)	2 (1.27)
Eye disorders	2675 (7.64)	152 (5.11)	109 (18.51)	6 (8.22)	11 (7.01)
Respiratory disorders	2320 (6.62)	402 (13.51)	58 (9.85)	4 (5.48)	1 (0.64)
Renal and urinary disorders	797 (2.28)	167 (5.61)	2 (0.34)	0 (0.00)	0 (0.00)
Reproductive and breast disorders	1284 (3.67)	72 (2.42)	5 (0.85)	1 (1.37)	1 (0.64)
Vascular disorders	921 (2.63)	205 (6.89)	9 (1.53)	2 (2.74)	2 (1.27)
Hepatobiliary disorders	858 (2.45)	136 (4.57)	6 (1.02)	1 (1.37)	0 (0.00)
Blood and lymphatic disorders	1391 (3.97)	476 (16.00)	5 (0.85)	1 (1.37)	0 (0.00)
Metabolism and nutrition disorders	1242 (3.55)	194 (6.51)	3 (0.51)	4 (5.48)	1 (0.64)
Neoplasms	817 (2.33)	915 (30.73)	28 (4.75)	5 (6.85)	0 (0.00)
Cardiac disorders	594 (1.70)	260 (8.74)	2 (0.34)	0 (0.00)	1 (0.64)
Ear and labyrinth disorders	562 (1.60)	30 (1.01)	0 (0.00)	0 (0.00)	0 (0.00)
Congenital disorders	625 (1.78)	46 (1.55)	7 (1.19)	6 (8.22)	0 (0.00)
Endocrine disorders	240 (0.69)	14 (0.47)	2 (0.34)	0 (0.00)	0 (0.00)
Social circumstances	216 (0.62)	8 (0.27)	3 (0.51)	0 (0.00)	0 (0.00)
Surgical procedures	1089 (3.11)	38 (1.28)	6 (1.02)	5 (6.85)	0 (0.00)
Product issues	41 (0.12)	35 (1.18)	0 (0.00)	1 (1.37)	1 (0.64)

**Table 5:** Statistically significant ROR values for Isotretinoin vs. other retinoids (95% CI).

SOC	Comparator	ROR	CI Low	CI High
Blood and lymphatic system disorders	TotalTRN	0.28	0.25	0.32
	Tretinoin	0.22	0.19	0.24
	Adapalene	4.83	2.0	11.67
Cardiac disorders	TotalTRN	0.23	0.2	0.27
	Tretinoin	0.18	0.15	0.21
Ear and labyrinth disorders	TotalTRN	2.05	1.42	2.96
	Tretinoin	1.6	1.11	2.32
Eye disorders	Tretinoin	1.54	1.3	1.82
	Adapalene	0.36	0.29	0.44
Gastrointestinal disorders	TotalTRN	3.88	3.5	4.31
	Tretinoin	3.61	3.22	4.05
	Adapalene	4.71	3.56	6.24
	Tazarotene	4.48	2.06	9.78
	Trifarotene	10.19	4.77	21.75
General disorders and administration site conditions	TotalTRN	0.66	0.61	0.71
	Adapalene	0.52	0.44	0.63
	Tazarotene	0.56	0.33	0.93
	Trifarotene	0.37	0.27	0.51
Hepatobiliary disorders	TotalTRN	0.64	0.54	0.77
	Tretinoin	0.52	0.44	0.63
	Adapalene	2.44	1.09	5.47
Immune system disorders	TotalTRN	0.19	0.16	0.23
	Tretinoin	0.33	0.26	0.43
	Adapalene	0.05	0.04	0.07
Infections and infestations	TotalTRN	0.65	0.59	0.72
	Tretinoin	0.55	0.49	0.61
Injury, poisoning and procedural complications	Tretinoin	1.13	1.02	1.26
Investigations	Tretinoin	1.29	1.14	1.45
	Adapalene	7.47	4.21	13.24
Metabolism and nutrition disorders	TotalTRN	0.65	0.56	0.76
	Tretinoin	0.53	0.45	0.62
Musculoskeletal and connective tissue disorders	TotalTRN	2.89	2.5	3.35
	Tretinoin	2.49	2.13	2.91
	Adapalene	6.0	3.59	10.02
Neoplasms	TotalTRN	0.07	0.06	0.08
	Tretinoin	0.05	0.05	0.06
Nervous system disorders	TotalTRN	0.68	0.62	0.74
	Tretinoin	0.57	0.52	0.63
Pregnancy, puerperium and perinatal conditions	Tretinoin	1.99	1.68	2.37
	Adapalene	2.68	1.73	4.15
	Tazarotene	0.18	0.11	0.29
Product issues	TotalTRN	0.12	0.08	0.19
	Tretinoin	0.1	0.06	0.15
Psychiatric disorders	TotalTRN	11.96	10.11	14.14
	Tretinoin	10.67	8.91	12.77
	Adapalene	20.9	16.38	26.67
Renal and urinary disorders	TotalTRN	0.5	0.42	0.59
	Tretinoin	0.39	0.33	0.46
Reproductive system and breast disorders	Tretinoin	1.53	1.21	1.95
	Adapalene	4.44	2.94	6.71
Respiratory, thoracic and mediastinal disorders	TotalTRN	0.51	0.46	0.56
	Tretinoin	0.45	0.41	0.51
Skin and subcutaneous tissue disorders	TotalTRN	0.69	0.64	0.75
	Tretinoin	1.46	1.3	1.63
	Adapalene	0.11	0.08	0.15
	Trifarotene	0.09	0.05	0.17
Social circumstances	TotalTRN	2.13	1.16	3.92
	Tretinoin	2.3	1.14	4.67
Surgical and medical procedures	TotalTRN	2.45	1.84	3.27
	Tretinoin	2.48	1.79	3.44
Vascular disorders	TotalTRN	0.44	0.38	0.52
	Tretinoin	0.36	0.31	0.43

ROR, reporting odds ratio; CI, confidence interval; SOC, system organ class, TotalTRN = pooled topical retinoids (tretinoin, adapalene, tazarotene, trifarotene). RORs compare isotretinoin with the specified comparator; ROR > 1 indicates over-reporting and ROR < 1 under-reporting for isotretinoin versus the comparator. Statistically significant denotes 95% CIs that do not include 1. Only statistically significant comparisons are shown; therefore, not all SOC–comparator pairs appear in the table. Across comparators, the strongest positive signals were observed for psychiatric, gastrointestinal, and musculoskeletal disorders.

## Data Availability

The original contributions presented in the study are included in the article; further inquiries can be directed to the corresponding authors.

## Abbreviations

The following abbreviations are used in this manuscript:

**ADR**	**Adverse drug reaction**
**ADRreports**	European Medicines Agency (EMA) web portal providing aggregated EudraVigilance data on suspected adverse drug reactions
**CI**	Confidence interval
**COVID-19**	Coronavirus disease 2019
**EEA**	European Economic Area
**EMA**	European Medicines Agency
**EU**	European Union
**EudraVigilance**	European Union pharmacovigilance database for suspected adverse drug reactions (European Union Drug Regulating Authorities Pharmacovigilance)
**GI**	Gastrointestinal
**ICSR**	Individual case safety report
**MedDRA**	Medical Dictionary for Regulatory Activities
**NAMCS**	National Ambulatory Medical Care Survey
**PT**	Preferred terms
**RAR**	Retinoic acid receptor
**RAR-α**	Retinoic acid receptor alpha
**RAR-β**	Retinoic acid receptor beta
**RAR-γ**	Retinoic acid receptor gamma
**ROR**	Reporting odds ratio
**SOC**	System organ class
**TotalTRN**	Pooled group of all topical retinoids (tretinoin, adapalene, tazarotene, trifarotene)
